# Technology-related behavioral addiction profiles and mental health in teachers

**DOI:** 10.3389/fpsyt.2026.1819456

**Published:** 2026-05-22

**Authors:** Yolanda Ruiz-Ordoñez, Elena Gervilla, Albert Sesé

**Affiliations:** 1 Department of Psychology, Valencia Catholic University Saint Vincent Martyr, Valencia, Spain; 2 Department of Psychology, University of the Balearic Islands, Palma de Mallorca, Spain; 3 Statistical and psychometric procedures applied to Health Sciences (PSICOMEST) Research Group, Fundacio Institut d’Investigacio Sanitaria Illes Balears, Palma, Spain

**Keywords:** teacher mental health, anxiety and depression, problematic internet and technology use, online vulnerability, behavioral addictions, compulsive buying, latent profile analysis

## Abstract

**Introduction:**

Teachers' mental health is increasingly challenged by ubiquitous digital demands, yet little is known about how specific constellations of online behaviors and compulsive buying cluster in this key professional group or how they map onto psychiatric symptomatology.

**Methods:**

This cross-sectional study examined problematic use of the internet, mobile phones, instant messaging, social networking sites and videogames, together with compulsive buying, in 1,043 in-service teachers from all educational stages in Spain. Participants completed validated self-report measures of problematic ICT use, compulsive buying, anxiety, depression and online vulnerability. Latent profile analysis combined with k-means clustering identified homogeneous behavioral profiles, and differences in affective symptoms and sociodemographic characteristics were tested using analysis of variance and chi-square tests.

**Results:**

Four distinct profiles emerged. A low-risk group showed minimal problematic ICT use or compulsive buying and very low levels of anxiety, depression and online vulnerability. Two intermediate groups were characterized by predominantly ICT-related risk or predominantly compulsive buying, each showing specific and clinically relevant patterns of emotional symptoms. A high-risk group, overrepresented by younger and predominantly female teachers, displayed concurrent problematic ICT use and compulsive buying together with markedly elevated anxiety, depression and online vulnerability, accounting for a large share of the variance in mental health outcomes. Younger teachers were consistently more likely to belong to the high-risk profiles that carried the greatest psychological burden.

**Discussion:**

These findings reveal substantial and clinically meaningful heterogeneity in digital and consumer risk behaviors among teachers and point to a subgroup with a profile compatible with behavioral addictions and significant psychiatric distress. The study underscores the importance of integrating digital-behavior screening into occupational mental health assessment in education and supports the development of targeted, profile-based interventions in digital literacy, emotion regulation and identity reinforcement to protect teachers' mental health in technology-rich environments.

## Introduction

The rise of digital culture marks an evolution toward an increasingly hyperconnected global society. Today, 64% of the world’s population are active social media users, 70.3% use mobile phones (1), and 68% have internet access, with European rates ranging from 87% to 92% (2). This technological shift has accelerated in the wake of the COVID-19 pandemic, particularly in the academic context, where educators have been compelled to acquire new digital competencies to meet the demands of a rapidly changing society (3). ICTs play an adaptive and significant role in education (4), and their use, along with smartphones, has been shown to positively impact personal, social, and professional domains by expanding opportunities for information and communication (5).

However, alongside these benefits, excessive and problematic ICT use has emerged as a concern, as it can lead to compulsive behaviors with social, psychological, and occupational consequences (6, 7). Compulsivity is a central construct in the study of behavioral addictions, though recent meta-analytic evidence suggests that the relationship between compulsivity and addictive behaviors is not necessarily unidirectional or causal (8). While the prevalence of problematic internet use in Europe is lower than in the United States, with Spain reporting the lowest rate among European countries at 2.1%, the problematic use of specific online services, such as social networks, videos, and email, remains a relevant issue (9). Even with these relatively low prevalence rates, compulsive behaviors associated with ICT use can still have negative repercussions for well-being. Notably, some studies suggest that digital disconnection, particularly from social media, does not necessarily enhance well-being or lead to significant changes in how individuals feel (10).

Against this broader backdrop of problematic ICT use, the concept of internet addiction has gained increasing attention. The definition of internet addiction remains a subject of debate (4), but it is generally understood as a compulsive-impulsive disorder characterized by excessive use and emotional consequences (11). The amount of time spent online is a key factor: some studies report that 94.7% of those who use the internet for only one hour per day do not show problematic use, compared to 16.4% among those who spend more than 10 hours daily online (12). Other research proposes thresholds of 8.5 hours per week for problematic use and over 21.5 hours per week for addiction (13). The prevalence of internet addiction in adults up to 35 years old has been estimated at 27.1% (14), and smartphone addiction is highest among those aged 20 to 24, followed by the 35 to 50 age group (15). In Spain, 23.67% of individuals over 17 years report smartphone dependence (16). While some studies suggest higher internet addiction among married individuals with higher education (12), the risk profile more commonly includes young people with lower incomes, single status (17), living with family (18), and frequent use of social networks (13). Additionally, personal and social difficulties, as well as negative emotions when unable to access the internet, are characteristic of addictive behavior (19).

Most of this evidence has been obtained in general or clinical populations, and research on problematic ICT use among professionals has primarily focused on the health sector. Notably, no severe internet addictions have been detected among veterinarians (20), whereas among health professionals, prevalence rates reach 79%, with 41% classified as moderate and 2% as severe addiction. A study of students and professionals from diverse fields (medicine, nursing, engineering, law, arts, commerce) found that 57.7% of those under 30 exhibit internet addiction, compared to 26.3% of those over 30, with younger individuals displaying less emotional attachment and mood impact (11). Within the health professions, social network addiction is more prevalent among those aged 18 to 30, who are single, have less than three years of experience, and report lower income satisfaction (21). The primary motivation for internet dependency in these groups is social connectivity (22), and sharing personal information on social networks further increases the risk of addiction (11).

Despite the predominance of research on adolescents and health professionals, the teaching profession itself has received comparatively little attention, even though teachers are intensive users of technology, social media, and the internet in their daily work (6, 23). Studies with pre-service teachers indicate that 21.4% spend between three and five hours daily on social media, placing them at risk for addiction (24). Recent findings suggest that 36.3% of future teachers have internet addiction problems (25), while others estimate that 28% are at risk and 8% have pathological addiction. Notably, using the internet for research purposes is associated with lower addiction risk and greater professional competence (26). Technology addiction in this group is more prevalent among men (27), particularly for online gaming (28), but can also involve smartphone use. The risk profile for ICT addiction among future teachers is more common among those with higher economic status, parents with university degrees, retired mothers, and parents employed in the public sector (29).

Building on this evidence in pre-service teachers, recent studies have begun to examine problematic ICT use among in-service teachers. Among in-service teachers, 10% use the internet for more than five hours daily, and 7% of secondary school teachers are at risk. While teachers use the internet for both work and personal purposes (gaming, entertainment, etc.) (30), using it for personal reasons increases the risk of addiction (31). The risk profile for internet addiction among teachers includes being male, having fewer years of professional experience, being under 35, using the internet for more than five hours daily, being single, without children, using the internet for specific purposes (online gaming, chatting, dating, etc.), working in rural areas, having high incomes, and working part-time (23). Physical education teachers show lower rates of internet addiction compared to other specialties; those with problematic behaviors are more likely to be single, under 29, with less than nine years of work experience, working in rural areas, and perceiving higher monthly income (5). Despite a high awareness of the negative consequences of technology or internet addiction, most teachers do not consider it necessary to seek professional help (6).

Alongside problematic ICT use, compulsive buying, also known as oniomania, has emerged as another behavior of concern. This condition is mainly characterized by high impulsivity, low self-control in purchases, deterioration of work life, and negative consequences such as guilt (32). Compulsive buying can occur both physically and online, with higher risk (21%) and prevalence (3.8%) in physical purchases compared to online (11% risk, 2.9% prevalence) (33). Among health science teachers, 87.5% of women are more addicted to online shopping compared to 12.5% of men (34). The prevalence of addictive buying is 21% in women (35), and other risk factors include being a young adult and not having a partner (36).

Both internet addiction and compulsive buying are closely related to mental health and affective disorders, which further underscores their relevance in occupational groups such as teachers. In young adults, depression increases the prevalence of internet addiction (7), and high-risk groups are significantly more likely to have emotional disorders such as depression and anxiety (17). Individuals who use social networks to pass the time have higher depression scores than those who use them to seek information (13). Among future teachers, higher internet addiction is positively associated with anxiety (4). Numerous studies have demonstrated the relationship between internet addiction and psychological distress, including stress, anxiety, and depression (23), with depression being considered a predictor of this type of addiction in emerging adults (37). When differentiating types of addiction, depression and anxiety are higher among those with online gaming addiction (38), who also tend to be younger and lack conflicts at home or school (39).

Regarding social media addiction as a specific manifestation of problematic ICT use, although no gender differences have been observed, prevalence is higher among individuals aged 18 to 25, without children, single, and unemployed (13). There is also a clear relationship with mental disorders, as those with anxiety and depression show greater addiction (40). In fact, spending time on social networks increases both addiction and depression levels (13), and smartphone addiction is a predictor of anxiety and depression (41). However, among future teachers, the relationship with anxiety is lower compared to other student groups (42), such as engineering students, who have a higher percentage of severe addiction, possibly due to the intensive use of technology by their teachers (43). Furthermore, passive use of social networks is associated with compulsive online shopping (44). These behavioral patterns are not isolated phenomena; they are increasingly understood in relation to broader mental health outcomes.

Extending these findings, compulsive buying is more frequent among women and is associated with depression (45). Several studies link this behavior with emotional states and disorders, with materialism being connected to the emotional state of happiness derived from accumulating possessions (46). It is evident that problematic use of ICTs and compulsive buying are behaviors of interest that affect not only individuals but also their professional performance. In this regard, teachers represent a particularly vulnerable group that warrants specific study.

Therefore, the objective of this work is (1) to identify and characterize distinct profiles of online behavior and compulsive buying among teachers, and (2) to analyze how these behavioral profiles are associated with key affective variables, specifically anxiety, depression, and vulnerability. By integrating a person-centered approach, this study aims to deepen understanding of the heterogeneity within the teaching profession regarding digital habits and emotional well-being. Ultimately, by shedding light on these behavioral and emotional profiles, this research aspires to inspire new pathways for supporting teachers’ well-being and empowering them to guide and mentor students thoughtfully and resiliently in an ever-evolving world shaped by digital technology and artificial intelligence.

## Methods

### Procedure and participants

A cross-sectional study was conducted in Spain, across several regions, using intentional sampling, considering the different strata within the teaching population to ensure greater representativeness. The LimeSurvey online questionnaire, which included both psychometric measures and sociodemographic variables, was estimated to take 30 minutes to complete. To carry out the process, school principals were contacted, who gathered their teachers in a room with internet access. A team researcher explained the research objectives, data protection procedures, and instructions. Subsequently, the QR code for accessing the questionnaire was provided, where participants gave their informed consent. The data were anonymous and collected between December 2023 and February 2024. Ethical approval for this study was obtained from the Ethics Committee of the University of the Balearic Islands (code 365CER23) prior to data collection. The studies were conducted in accordance with the local legislation and institutional requirements. The participants provided their written informed consent to participate in this study.

### Measures

The behavior related to Information and Communication Technologies (ICTs) was measured using the MULTICAGE-TIC scale (47). It consists of 20 items and a six-level Likert scale ranging from never to always. It addresses issues related to problematic use of the Internet (items 1-4), mobile phones (items 5-8), video games (items 9-12), instant messaging (items 13-16), and social networks (items 17-20). The response is dichotomous (YES/NO), with affirmative responses indicating more problematic behavior. For each subscale, item 1 addresses the estimation of time spent, item 2 the estimation of significant people, item 3 the difficulty in developing this behavior, and item 4 the difficulties in voluntarily interrupting the behavior. The multivariate consistency of the complete test was α = .91.

Compulsive buying behavior was analyzed using the Compulsive Buying Scale (48). It consists of 13 items with a 5-level Likert scale and 3 subscales: tendency to spend (items 1, 2, 9, 10, 11, and 12), urgency to buy (items 3, 4, 5, and 8), and feelings of guilt (items 6, 7, and 13). The score range is from 13 to 65, with a Cronbach’s alpha of.88.

Vulnerability was measured using the Online Vulnerability Scale (49). It consists of 6 items with a 5-level Likert scale. Participants indicate the frequency with which they have encountered or witnessed situations of online vulnerability. Higher scores indicate greater vulnerability, with a Cronbach’s alpha of.91.

Anxiety and depression were measured using the Spanish-adapted version of the General Health Questionnaire (GHQ-12) (50). This is a 12-item Likert-type scale with two subscales: 4 items to measure anxiety and 8 items for depression. Higher scores indicate lower psychological well-being. For both subscales, the validation indicated a Cronbach’s alpha of.75.

### Statistical analysis

Descriptive statistics were calculated to characterize the sample in relation to demographics, online behaviors, vulnerability, anxiety and depression. Latent profile analysis (LPA) was performed with the R package *snowRMM* (51) to identify online behavior profiles among teachers. Following prior recommendations (52–55), five statistics were used to determine the number of latent profiles: Log-likelihood (logLik), Akaike information criterion (AIC), Bayesian information criterion (BIC), sample-size adjusted Bayesian information criterion (SABIC), and the Entropy. While there are no formal cut-offs, lower values of AIC and BIC indicate better model fit (53). Like the BIC, the SABIC balances model fit (as measured by the log-likelihood) with model complexity (the number of estimated parameters), but its penalty is less strict when sample sizes are moderate or small. Entropy values which range from 0 to 1 represent the accuracy of classification, and it is generally considered that values >.80 imply high accuracies. Researchers also recommended that the smallest class should comprise at least 5% of the total sample (56).

K-means clustering using the Hartigan-Wong algorithm was performed after LPA as a complementary approach to compare the results obtained from these two distinct clustering techniques. K-means is a non-model-based, distance-driven algorithm that partitions the data into k clusters by minimizing within-cluster sum of squares based on Euclidean distances. The Hartigan-Wong variant iteratively reassigns individual data points to alternative clusters whenever such moves improve cluster compactness, updating cluster centers accordingly for greater efficiency and stability (centroid values in z scores). This comparison is recommended in the literature to validate and triangulate findings (57), as LPA and k-means may yield different group structures due to their underlying assumptions and sensitivity to data characteristics. To determine the optimal number of clusters, we relied on established evaluation methods such as the elbow method, which examines the reduction in within-cluster sum of squares as the number of clusters increases. A set of 2-, 3-, and 4-cluster solutions was estimated, emulating those obtained from the LPA. K-means clustering was implemented using the *snowCluster* module in Jamovi (58).

Lastly, a set of one-way analysis of variance (ANOVA) and a set of chi-square analyses were carried out to examine differences between clusters in continuous (e.g., depression, anxiety, vulnerability) and categorical variables (e.g., gender), respectively. Prior to conducting the ANOVA analyses, the assumptions of normality and homogeneity of variances were examined using the Kolmogorov-Smirnov test and Levene’s test, respectively. Effect size was evaluated with Eta squared, Cohen’s d and Cramer’s V, as appropriate. All analyses were performed with jamovi version 2.6 software (59).

## Results

### Descriptive analysis

A total of 1,043 in-service teachers participated in the study (71.2% women, *n* = 743). The mean age was 45.5 years (*SD* = 9.9). No missing values were found in the database. Detailed sociodemographic and professional characteristics of the sample (including marital status, children, type of institution, educational stage taught, tutor role, sick leave, religious beliefs, and social volunteering) are presented in **Table 1**.

**Table 1:** Sample characteristics of participating teachers (N = 1,043).

Variable	Category	n	%
Sex	Male	300	28.8
	Female	743	71.2
Marital status	Single	261	25
	Married	689	66.1
	Divorced	67	6.4
	Widowed	12	1.2
	Separated	14	1.3
Has children	Yes	722	69.2
	No	321	30.8
Type of institution	Public/State-subsidized	803	77
	Private	240	23
Educational stage	Early childhood	125	12.7
	Primary education	400	40.5
	Secondary education	337	34.1
	High school	97	9.8
	University	3	0.3
	Vocational Training	25	2.5
Tutor role	Yes	576	58.4
	No	411	41.6
Sick leave in the last 2 years	Yes	376	36
	No	667	64
Religious beliefs	Yes	977	93.7
	No	66	6.3
Social volunteering	Yes	476	45.6
	No	567	54.4

**Table 2** shows the correlation between the measurements. All correlations were statistically significant. Correlations between age and all other measurements were negative.

**Table 2:** Correlation matrix between all measurements.

Variables	1	2	3	4	5	6	7	8	9	10	11
1. Internet	—										
2. Mobile	.81***	—									
3. Videogames	.38***	.31***	—								
4. Messaging	.71***	.80***	.24***	—							
5. Networks	.67***	.67***	.27***	.69***	—						
6. Prop_ spend	.39***	.41***	.18***	.43***	.40***	—					
7. Urgency	.30***	.34***	.15***	.38***	.34***	.76***	—				
8. Guiltiness	.37***	.40***	.18***	.41***	.35***	.70***	.68***	—			
9. Anxiety	.10**	.11***	.09**	.13***	.10**	.07*	.09**	.06 ns	—		
10. Depression	.16***	.15***	.07*	.14***	.14***	.12***	.16***	.18***	.31***	—	
11. Vulnerability	.33***	.27***	.17***	.27***	.29***	.20***	.17***	.19***	.07*	.12***	—
12. Age	-.37***	-.34***	-.14***	-.33***	-.44***	-.25***	-.17***	-.16***	-.07*	-.08*	-.17***

ns, non-significant, * = p<.05, ** = p<.01, ***=p<.001.

### Identification of online behavior latent profiles

Model fit indices showed that the two-profile solution had a LogLik of -20894, a SABIC of 41883, and an entropy of .89. The three-profile solution improved these values, with a LogLik of -20344, a SABIC of 40815, and an entropy of .90. The four-profile solution demonstrated the best fit, with a LogLik of -19998, a SABIC of 40157, and an entropy of .91. Both AIC and BIC also decreased as the number of profiles increased, further supporting the improved fit of more complex models. Based on these indices, the four-profile model was selected as the optimal solution, as it provided the best balance between model fit and classification reliability (53). Therefore, based on these indices, the 4-profile solution would be preferred, provided that the resulting profiles are theoretically meaningful and adequately sized (60).

**Figure 1:**
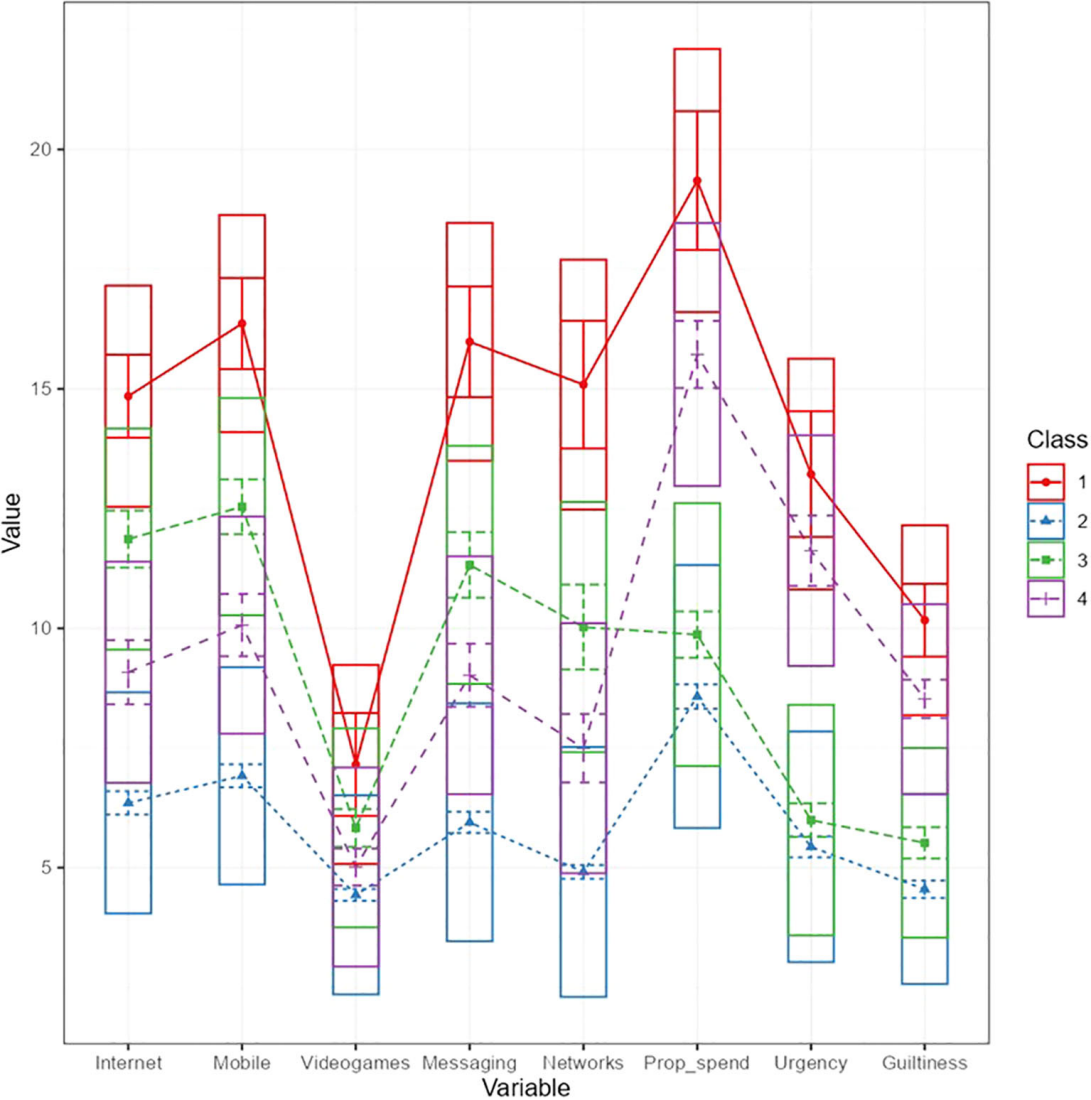
Latent profile plot assuming equal variances and no covariances (model 1; 4 profiles).

**Figure 2:**
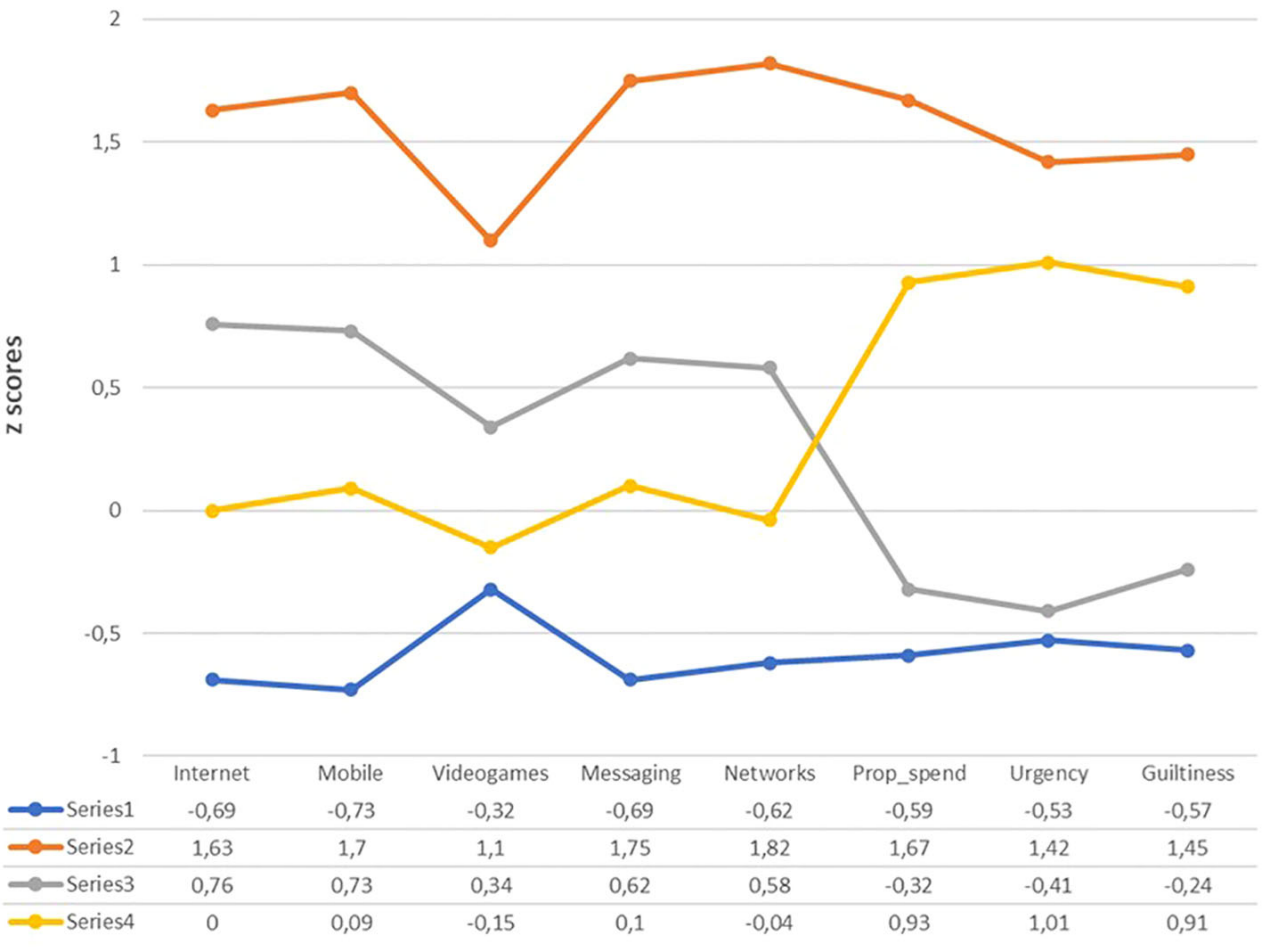
Clusters’ plot using the Hartigan-Wong algorithm (4 clusters).

**Figure 1** shows the LPA plot with the boxplots of the four latent profiles and the lines assuming equal variances and no covariances between them. And the **Figure 2** displays the Cluster’s plot obtained after implementing the k-means procedure with the Hartigan-Wong algorithm and the centroids of the variables conforming the clusters (standardized values).

**Figure 2** displays four distinct teacher profiles based on problematic ICT use and buying compulsivity:

Cluster 1 (LICT/B: Low Problematic ICT Use and Buying Compulsivity) shows consistently negative z-scores across all variables, with particularly low values in mobile use (-0.73) and internet use (-0.69), indicating minimal problematic behaviors in both domains.

Cluster 2 (HICT/B: High Problematic ICT Use and Buying Compulsivity) presents uniformly elevated z-scores, peaking in social network use (1.82) and messaging (1.75), reflecting high-risk behavior across all dimensions.

Clusters 3 and 4 reveal complementary patterns: Cluster 3 (RICT/LB) exhibits moderately positive z-scores in ICT use (particularly internet and mobile) but negative scores in buying behaviors, while Cluster 4 (AICT/HB) shows the inverse-near-average ICT use but distinctly elevated buying compulsivity scores, particularly in urgency (1.01).

These profiles suggest that Clusters 1 and 2 represent opposite ends of a severity continuum, while Clusters 3 and 4 demonstrate that problematic technology use and compulsive buying can manifest independently of each other, indicating potentially distinct underlying mechanisms.

**Figure 3:**
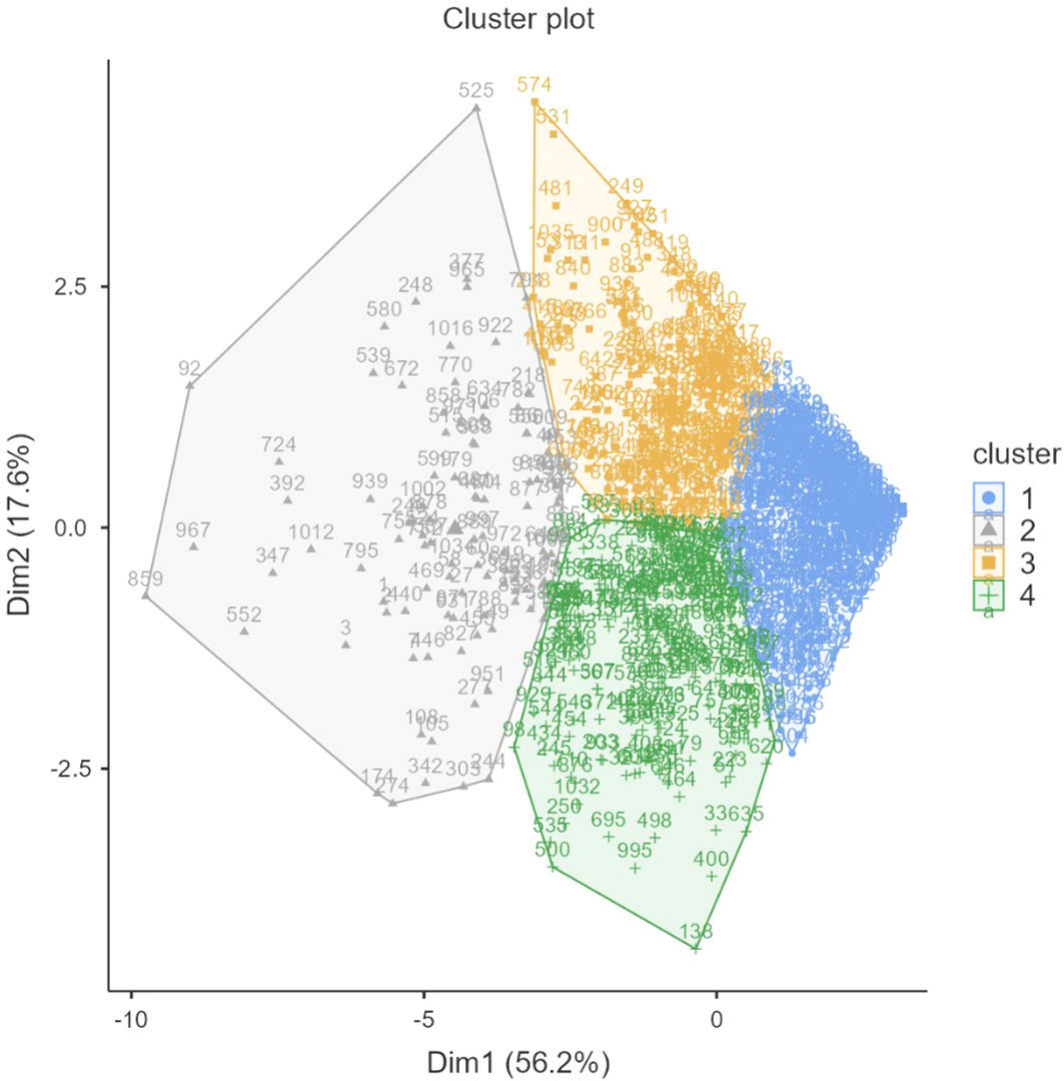
Cluster plot with the distribution of individuals across four clusters in the space defined by the first two principal dimensions.

Both LPA and k-means clustering identified four groups. LPA captures within-profile variability and overlap, whereas k-means produces more sharply defined clusters with greater mean differences across variables. This clearer separation in the k-means solution facilitates subsequent group comparisons. **Figure 3** displays participants’ locations on the first two principal dimensions, with clusters indicated by different colors.

There were statistically significant differences between clusters in online behaviors (**Table 3**) using ANOVA. Kolmogorov-Smirnov’s tests indicated no substantial deviations from normality and Levene’s tests supported the homogeneity of variances assumption. All clusters differed significantly from one another on each subscale, except for video game use (where Clusters 1 and 4 did not differ significantly) and urgency to buy (where Clusters 1 and 3 did not differ significantly). The highest levels of problematic ICT use were found in Clusters 2 and 3, while the highest compulsive buying scores were observed in Clusters 2 and 4. Overall, 52.7% of teachers exhibited some form of problematic or compulsive behavior.

**Table 3:** Participants’ characteristics by cluster with ANOVA and *post-hoc* comparisons.

Variables	Total sample (n=1043)	a Cluster 1 (n=493, 47.3%) *LICT/B*	b Cluster 2 (n=99, 9.5%) *HICT/B*	c Cluster 3 (n=234, 22.4%) *RICT/LB*	d Cluster 4 (n=213, 20.4%) *AICT/HB*	F	Size effect
Internet	8.74 (3.68)	6.21^bcd^ (1.90)	14.75^acd^ (2.99)	11.55^abd^ (2.64)	8.77^bcd^ (2.42)	535.79***	.61
Mobile	9.49 (3.82)	6.71^bcd^ (2.05)	16.01^acd^ (2.69)	12.29^abd^ (2.32)	9.84^bcd^ (2.42)	646.60***	.65
Videogames	5.07 (2.24)	4.35^bc^ (1.04)	7.55^acd^ (3.93)	5.83^abd^ (2.68)	4.73^bc^ (1.50)	83.61***	.20
Messaging	8.50 (4.0)	5.75^bcd^ (1.77)	15.49^acd^ (3.99)	11.00^abd^ (2.93)	8.90^abc^ (2.60)	530.90***	.61
Networks	7.33 (4.05)	4.80^bcd^ (1.42)	14.72^acd^ (4.25)	9.68^abd^ (3.47)	7.19^abc^ (2.80)	470.50***	.58
Propensity to spend	11.01 (4.54)	8.35^bcd^ (2.21)	18.58^acd^ (4.32)	9.56^abd^ (2.25)	15.25^abc^ (3.42)	588.60***	.63
Urgency	7.29 (3.77)	5.31^bd^ (1.91)	12.66^acd^ (3.80)	5.73^bd^ (2.01)	11.11^abc^ (3.16)	465.31***	.57
Guiltiness	5.93 (2.76)	4.35^bcd^ (1.59)	9.94^acd^ (2.35)	5.27^abd^ (1.92)	8.45^abc^ (2.17)	404.59***	.54

Mean (SD); ***, p<.001; ^a,b,c,d^, Significant post-hoc comparisons between clusters.

A significant association was found between cluster membership and sex [χ²(3) = 34.48, p <.001, V = .18]. Females (n = 741) were more frequently represented than males (n = 298) in Clusters 2 (11% vs. 6%) and 4 (24% vs. 12%), while males were more prevalent in Clusters 1 (50% vs. 46%) and 3 (32% vs. 19%). There was also a significant relationship between clusters and age [F(3, 1035) = 53.37, p <.001, η² = .13]. Cluster 1 had the highest mean age (M = 48.77, 95% CI [47.95, 49.59]), followed by Clusters 4 (M = 44.54, 95% CI [43.30, 45.79]) and 3 (M = 43.03, 95% CI [41.85, 44.22]), with Cluster 2 having the lowest mean age (M = 37.20, 95% CI [35.38, 39.03]). All *post-hoc* comparisons were statistically significant except between Clusters 3 and 4, indicating similar age distributions for these two groups.

### Relationship of online behavior profiles and anxiety, depression and vulnerability

As shown in **Table 4**, teachers in the ‘low’ profile (Cluster 1) scored lowest in vulnerability, anxiety, and depression, while those in the ‘high’ profile (Cluster 2) scored highest on these indicators. Teachers in the ‘average’ profile (Cluster 4) reported intermediate values across all three measures. Cluster 1 was significantly the least vulnerable (12.60), indicating better mental health. Cluster 2 was the most vulnerable (18.18) and had the highest depression scores (20.36), as well as significantly higher anxiety (11.21) compared to Clusters 1 and 4, both of which had low compulsive buying behavior. Clusters 3 and 4 showed intermediate scores for vulnerability, anxiety, and depression relative to Cluster 2. Notably, the anxiety score for Cluster 3 (10.81) did not differ significantly from any other cluster.

**Table 4:** Cluster differences in vulnerability, anxiety, and depression with ANOVA and *Post-Hoc* means comparisons.

Variables	Total sample	a Cluster 1 LICT/B	b Cluster 2 HICT/B	c Cluster 3 RICT/LB	d Cluster 4 AICT/HB	F	Effect size
Vulnerability	14.1	12.60^bcd^	18.18^acd^	15.35^ab^	14.51^ab^	29.97***	.08
Anxiety	10.7	10.60^b^	11.21^ad^	10.81	10.66^b^	6.36***	.02
Depression	19.6	19.36^bc^	20.36^acd^	19.75^ab^	19.70^b^	10.78***	.03

***, p<.001; ^a,b,c,d^, Significant post-hoc comparisons between clusters.

## Discussion

This study aimed to identify behavioral profiles related to online activity and compulsive buying among teachers, and to analyze their associations with anxiety, depression, and vulnerability. The results revealed four distinct patterns of online behavior and buying conduct within this population, with notable demographic differences: female participants predominated in Clusters 2 and 4, while male participants were more represented in Clusters 1 and 3. By focusing specifically on teachers, a group often underrepresented in behavioral addiction research despite facing important digital stressors, these findings contribute valuable insight to the literature on behavioral addictions and digital health (22, 61). Importantly, as teachers serve as key gatekeepers and role models for children and adolescents, their own online behaviors and susceptibility to compulsive buying may have broader implications, potentially influencing the digital habits and emotional well-being of the students in their care (62).

A closer examination of each profile illuminates the psychological and demographic characteristics that differentiate these groups. Cluster 1 (LICT/B), composed mainly of older participants (*M* = 48.77 years), is characterized by low vulnerability, anxiety, and depression, as well as minimal problematic ICT use and compulsive buying. This group likely benefits from strong social support and emotional stability, suggesting that reinforcing these protective factors may be beneficial. In contrast, Cluster 2 (HICT/B), with a higher proportion of younger and female teachers (*M* = 37.20 years), exhibits the highest levels of problematic ICT use, compulsive buying, vulnerability, anxiety, and depression. This profile aligns with prior research linking younger age and female gender to greater risk for behavioral addictions and emotional distress (12, 23, 63). Interventions for this group should focus on emotion regulation, digital boundaries, and social skills training (46, 64).

While Clusters 1 and 2 represent opposite ends of the risk continuum, Clusters 3 and 4 reveal more nuanced patterns. Cluster 3 (RICT/LB) presents moderately high ICT use and some emotional distress, particularly related to depression, but low compulsive buying. This pattern suggests a need for targeted digital literacy and mental health support, especially given the association between smartphone use and addictive behaviors (65, 66). Cluster 4 (AICT/HB) is distinguished by high compulsive buying and average ICT use, with indications that ego-driven motives and the pursuit of social recognition may underlie these behaviors (32, 67). For this group, interventions addressing self-esteem and personal values may be particularly effective.

The mental health burden associated with these profiles is most pronounced in Cluster 2, which obtained the highest scores in vulnerability (18.18), depression (20.36), and anxiety (11.21), a pattern consistent with studies linking depressive symptoms to internet addiction (9, 65), although other research suggests that depression may be the least influential variable in this context (25, 68). While it remains unclear whether the causal relationship is unidirectional or mutually reinforcing (69), the prevalence of internet addiction has been reported to reach as high as 36% among individuals with depression (70). Recent studies have shown that anxiety and depression can be consequences of problematic internet use, with individuals experiencing more severe depressive symptoms being more prone to addiction (19). Moreover, the presence of higher anxiety levels, particularly in Cluster 2 where female participants are more represented (4), is associated with an increased risk of internet addiction (23). These findings reinforce existing evidence linking anxiety and depression with problematic social media use (40, 71). Addictive social media behaviors may emerge as coping mechanisms to alleviate symptoms of anxiety and depression (21). Although identifying the specific platforms used would be valuable, particularly since Instagram has been associated with higher levels of addictive behavior, the amount of time spent on social media has been shown to correlate positively with depression (13).

Beyond mental health outcomes, the high compulsive buying scores observed in Clusters 2 and 4 suggest that these profiles exhibit high levels of impulsivity and a desire for immediate gratification, which may drive the need to purchase. However, this behavior is often followed by negative emotional states, such as guilt (45). In addition to impulsivity or lack of self-control, materialism, boredom, and negative emotional states can also contribute to this type of online (72) and offline behavior. It is possible that the motivation for purchasing stems from the desire to alleviate negative feelings (35), though this may eventually lead to feelings of guilt, which are more closely related to depression and stress. Anxiety could also serve as a predictive variable for compulsive buying (73).

These behavioral and emotional patterns take on particular significance when considered in light of the professional role of teachers. The implications of these findings may be particularly relevant given the critical role teachers play as gatekeepers of children’s digital experiences and emotional development. Teachers not only model technology use and self-regulation, but also shape the classroom environment and influence students’ attitudes and behaviors toward digital media (74, 75). Research demonstrates that teachers’ digital competence and their own technology-related behaviors can significantly impact both their well-being and student learning outcomes (76). Furthermore, teachers’ awareness and management of their digital practices are essential for guiding students in healthy technology use, fostering emotional growth, and preventing negative impacts such as impaired social skills, reduced academic achievement, and emotional dysregulation (75).

The interpretation of these findings must be considered alongside several methodological limitations. It is important to note its cross-sectional design and the use of a purposive sample. Additionally, the data were self-reported, which may introduce bias. Incorporating a tool for measuring stress would have been beneficial, given its strong relationship with teachers’ mental health (61), as well as with internet and smartphone addiction (77, 78). The lack of differentiation between online and offline compulsive buying is another limitation, as it may obscure platform-specific dynamics (32).

Notwithstanding these limitations, the significance of this research lies not only in the scarcity of studies addressing problematic ICT use and compulsive buying among teachers, but also in its contribution to understanding how these behaviors intersect with mental health and professional functioning in educational contexts. By identifying distinct behavioral profiles and their associations with anxiety, depression, and vulnerability, this study provides novel evidence that teachers are not a homogenous group with respect to digital risks, but rather display diverse patterns that may require tailored support and intervention strategies. The findings underscore that teachers’ behavioral health, especially regarding digital habits and emotional regulation, is intimately linked to their effectiveness as affective and behavioral gatekeepers for children and adolescents.

Beyond their descriptive value, these results carry important practical and theoretical implications. Practically, these results highlight the urgent need for schools and policymakers to implement targeted prevention and intervention programs that address both digital self-regulation and emotional well-being among teachers. Training in coping strategies, digital literacy, and emotional intelligence should be prioritized to mitigate the risk of behavioral addictions and to foster a healthier educational environment. Furthermore, schools should consider ongoing professional development and peer support networks to reinforce positive digital habits and psychological resilience. The role of school leadership is also crucial in creating a supportive climate that recognizes and addresses the digital and emotional challenges faced by teachers, facilitating access to mental health resources and promoting a culture of openness regarding behavioral health concerns. In addition, the integration of technology policies that promote balanced and mindful use of digital tools can further support teachers in navigating the complexities of modern educational demands.

This research contributes to the literature on affective health by highlighting how occupational context shapes diverse patterns of emotional and behavioral risks within the teaching profession, emphasizing the multifaceted nature of these profiles beyond clinical or addictive behaviors. It also invites further exploration of the bidirectional relationship between technology use and emotional well-being, as well as the potential mediating effects of organizational culture and community support. By broadening our understanding of these dynamics, future studies can inform the development of more nuanced models that account for individual, contextual, and systemic factors influencing teachers’ digital and emotional health.

To that end, future research should prioritize longitudinal and mixed-methods designs to clarify causal relationships and the evolution of behavioral profiles over time. The inclusion of objective stress measures, as well as in-depth exploration of platform-specific behaviors and the impact of emerging digital trends, will be essential for developing more precise and effective interventions. Moreover, expanding research to diverse educational settings and cultural contexts will help to generalize findings and inform global strategies for promoting both teacher and student well-being in the digital age. Ultimately, supporting teachers’ mental health and digital competence is not only vital for their own professional fulfillment, but also for safeguarding the emotional and educational development of the students they serve. By investing in teacher well-being, educational systems can ensure a more resilient, adaptive, and thriving learning environment for all.

## Data Availability

The datasets presented in this study can be found here: https://doi.org/10.5281/zenodo.15448387. Further inquiries can be directed to the corresponding author/s.
